# American Dental Association

**Published:** 1893-01

**Authors:** 


### AMERICAN DENTAL ASSOCIATION.

(Continued from page 898.)

### DISCUSSION ON DR. BARRETT'S PAPER.

*Dr. Hunt.*—I would like to inquire whether it is possible to distinguish which molar is in use in the living animal. I do not doubt but that the first, second, or possibly the third molar could be distinguished in size; but when you get to the fourth, fifth, or sixth, is it possible to know, except by an examination of the skull, and even then without going into the cribs which contain the germs of the later teeth?

*Dr. Barrett.*—A number of teeth are there and may still remain there. That, of course, can be determined, and if I should find a tooth with eight, nine, or ten plates, I should consider it a third molar.

*Dr. Truman.*—I was interested in the paper, but it does not afford food for discussion. It is a statement of absolute facts. There was one point that Dr. Barrett positively asserted, that I do not feel is absolutely determined, and that is in regard to the three anterior molars. He affirms that they are not deciduous. I think authorities are not in unison on that question. I should like to have it stated on what ground this is determined. I understand, of course, that they have no successors in the sense of premolars, but still they seem to me to have a deciduous character.

*Dr. Thompson.*—This paper is somewhat elementary, and there is nothing to which I could take exception, except one point with regard to the incisors of the rodents. I think the functional difference is very great. They are developed for different purposes. The incisor of the elephant is developed, as we know, for uprooting trees and plants for food, and for weapons in battle, and it would not be possible for them to be used for the reduction or mastication of food, whereas those of the rodents are made for cutting up food. In regard to these specimens, I think the abnormity is produced naturally by lack of wear, and that it has a relation to the selection of food. It seems to me the lack of wear has not disposed of the preceding teeth in time for the new production of the succeeding teeth, and the new tooth has begun to be produced before the old one is out of the way, and therefore it interferes with the production of the first and second teeth. It seems to me Dr. Barrett is wrong in regard to the excessive wear in captivity and the restriction of the animal to particular foods,—foods which it would not get in freedom. I judge that this is altogether due to the lack of use in captivity.

*Dr. Peirce.*—I have been interested in the paper very much, and interesting papers afford food for thought. I have no criticisms to make, but I want to add a word or two to what has been said. The matter that is of importance to us is the fact that the arrangement of these plates of enamel on the teeth of animals indicates the excursion of the jaw. It is interesting to show the correspondence with the habits of the animal. Wherever we have the plates standing as these do, at right angles to the body of the tooth, we must have an antero-posterior motion of the jaw. It is the adaptation of the parts to use. Moving the jaw backward and forward brings these plates into their greatest use, while where we have the slight modification in the lower one of those two, there would be a slight excursion of the jaw. Where we have the plates standing in three different rows, we have not only an antero-posterior, but a lateral motion. The excursions of the jaw always correspond to the arrangement of the plates of enamel. It is a most beautiful adaptation of the parts to the use.

In regard to the case of Jumbo, I am inclined to coincide with Dr. Thompson that the second tooth commenced to form before the other had fully developed, and that caused an irregularity. The posterior part of the tooth is not formed at the time the anterior is. We have these plates added. So the want of room would have a tendency to throw this out. It would be interesting to know whether, with the arrangement of these plates in an irregular condition, Jumbo in using his teeth was known to modify at all the excursion of his jaws,—whether he had any other motion of the jaw than the normal.

*Dr. Truman.*—I would call Dr. Barrett's attention to the change in the form of the teeth from the mastodon to the elephant. It is an interesting fact that this was doubtless due to the change in the character of food from the period of the former, when this must have been coarse, as limbs of trees, to the finer grades in use by the modern proboscidium.

*Dr. Barrett.*—Dr. Truman says those teeth may be deciduous teeth. A deciduous tooth is one thrown off for the purpose of being succeeded by another. A deciduous tooth is one which belongs to an especially different dentition, and a different development of the teeth. For instance, in the diphyodonts we have different teeth. They are succeeded by teeth of another formation. In the elephant and all the proboscidiæ there is no such succession of teeth. They are simply shed, the one shed being succeeded posteriorly by another. There is no formation; there is no advancement beneath the deciduous tooth of a permanent tooth, consequently they are all of the same character. They differ in size, but that is all. They are identical in structure, and I do not see that they can be considered as deciduous. If the first molar be deciduous, the second can be considered deciduous too, and so on. We know that the structure of the tooth is that of permanent teeth, and therefore we may consider them all as permanent teeth, for they all have the same characteristics, and must either be all permanent or all deciduous.

In answer to Dr. Thompson: The incisors of elephants, he says, are not developed like those of the rodentia, and therefore would not be worn away, because the office is the same. That has nothing to do with it. The tooth being erupted, it may be put to any use. In all teeth of persistent pulps, whether of rodentia or proboscidiæ, the tooth will continue to grow and develop upon the line of the circle of its development, until it reaches its final size.

Regarding the wear of the teeth of Jumbo, I fully comprehend the weight of authority which may be against me, but I want to have my own opinion as far as I can. In its native state, the elephant lives upon succulent food; I mean that the green boughs will give much less attrition than the food of captivity. Consequently it must be the case that the wear of the teeth must be greater in the captive elephant than in its native state. Dr. Thompson, I think, said that the anterior plates were developed first. True. That sustains my position. The anterior plates in this case are very widely separated, unusually so. This would not be the case if it were due to pressure, which would tend to condense the plates. It is in the anterior plates that you find this wide separation of the enamel rings.

Did Jumbo have a different excursion of the jaw? I do not know, and we can hardly discover it; but I have examined the skull as perfectly as I could in the limited time I had, and as near as I could determine from the condyles of the jaw and the second bicuspid, it was no different from any other I ever saw. However, I cannot be positive. I hope to have the privilege of making sections of the jaw at some future time.

The evolution of the teeth from the mastodon to the elephant is the last point to which I wish to call your attention. I have here the teeth of a mastodon. Count the development of the tooth: the enamel covers the whole surface, but we have the dentine within the centre. In the course of development, by the wearing down of these eminences, what will be the shape and form of the enamel? Cut off the top of that until you reach the body of dentine, and you have the complete ring of the enamel that you find in the elephant. Separate the different denticles here, and they become dentinal plates, being worn down in the long process of development, and you have exactly the teeth of the elephant as they would be produced by the difference in the environments. Such a modification might be produced as will give the enamel this difference. In the body of the jaw the whole system is developed. We have an intermediate form in the diphyodonts.

*Dr. Peirce.*—Do you not recognize the fact that in the development of these teeth, if this animal were subjected to the same friction, we would have the same tooth? These are softer on account of the difference in food. These depressions are quite filled with cementum, and it is worn out.

*Dr. Barrett.*—The tooth is not developed in that way. It is developed from the enamel eminences. I cannot speak authoritatively, because I do not know. I will never have the opportunity of examining the animal in its native state; it is almost extinct. There is no more trace of any connection of cementum than there is on the enamel of the condyles.

Dr. W. B. Ames, chairman of Section I., "Prosthetic Dentistry, Chemistry, and Metallurgy," then read his report, as follows:

"Section I. has had very little matter offered this year. We have no papers to report. With the indulgence of the Association, Dr. George Evans will describe a process of enamelling the surfaces of gold crowns. As he is not present just now, we will offer the other matter first, which properly comes under the head of chemistry,—a new oxyphosphate, the oxyphosphate of copper,—which I have personally brought out in the last year, and only offer it as a new thing, for what there may be in it, and I will pass samples of it about, so you may see what the nature of it is. It is a mixture of black oxide of copper and phosphoric acid. It is almost the same as oxide of zinc, except that the black oxide of zinc is used. In addition to this, we want to recommend that Dr. Melotte, of Ithaca, give a clinic demonstrating the use of a new fusible metal, which fuses at a lower heat than those now in use; also a method of getting an absolutely accurate bite for bridge-work. He is prepared to show this if an opportunity is offered. We also want to offer a resolution before the Association to the following effect:

"WHEREAS, Section I. has been unable, in recent years, to obtain sufficient matter to make a proper report; and

"WHEREAS, It is the opinion of the Section that it is properly entitled to papers on crown- and bridge-work, and orthodontia; therefore, be it

"*Resolved*, That hereafter all papers on that subject be submitted to Section I."

Report received and resolution adopted.

*Dr. Low.*—Before Dr. Evans speaks I would like to ask a question in regard to the oxide of copper. We have seen the specimens, and I would like to know what advantages there are over the oxide of zinc.

*Dr. Ames.*—This is something that I offer because it is new, and possibly there is some value in it. The resulting compound is a very dense cement, and very hard. It seems to have a density and texture superior to any cement I have ever seen. Another advantage is that it can be used at a different consistency than other cements. Where we get best results from other cements in working by having them of a putty-like consistency, better results are obtained from this by using it in a fluid state. If it proves to have any permanency, which I think it has, the possibility of using it in this state is a decided advantage. It can be blown into a cavity, and allowed to become hard, and better results can be obtained from it. I have set crowns and bridges with it, and it allows of a crown or bridge being forced absolutely to its place.

*Dr. Low.*—How rapidly does it set, compared with the oxide of zinc?

*Dr. Ames.*—In the cold state it allows of considerable time for manipulation, but in the temperature of the mouth it crystallizes very rapidly, and must be worked quickly. There is a great difference in its qualities in winter and summer. I have found the cement prepared for winter to be hardly the proper thing for summer use.

*Dr. Smith.*—I would like to know if Dr. Ames has found it more irritant to the tissues than oxychloride.

*Dr. Ames.*—I think so. In some cases there was a marked irritation, and in others not.

*Dr. Smith.*—I have set crowns and bridges with it, and found it was quite irritating to the soft tissue, and it takes a much longer time to set. The great objection is its color. It is perfectly black, and produces discoloration. It has its use, however, as it becomes very dense and hard and insoluble. I am very glad to take the specimen, and I think it should have a place in our cabinets.

*Dr. Evans.*—In regard to the influence of heat in the mixing of cements,—the oxyphosphate of zinc and the cement presented by Dr. Ames,—it can be overcome in a very simple manner. Use as a slab for mixing a square 8-ounce bottle. Fill that with ice-water, and in the summer you will have about the same temperature to fix your cement with, on account of the cold given out by the ice, as you had in winter. In mixing oxyphosphate cement in winter, probably the cement will be at a temperature of about 65°. In summer, when it is 90°, there is a difference of about 35°, and the bottle is a very good thing to use. You can hold it in your left hand, and with the right mix the cement. I take an eight-ounce bottle, and place on one corner my acid, to the other side my powder; in the centre, between the two, my bridge or crown. I leave those in position. I go to my patient, and prepare the mouth, fix the napkins, and dry the teeth; then I take my slab and mix the cement. I insert it in the crowns or bridge, and after seeing that everything is right in the patient's mouth, I take up what cement I need, fix it in the tooth or cavities, quickly pick up the bridge, adjust it in position, remove the napkin, occlude the teeth, and everything is well. If you are interrupted, you can even stop for a moment.

In regard to the other matter, I did not come here intending to present it to the Association, but there seems to be so much interest taken in it that I will attempt to describe the method.

A plan by which the labial surface of upper first and second bicuspid gold crowns can be finished or enamelled is most desirable. This process possesses the advantage of forming a strong facing, and one that firmly adheres to the metal. It is as follows: A gold crown should be constructed, of a high grade of gold plate, nearly or entirely destitute of copper alloy, or it should be made of gold and platina crown metal. After the crown is fitted to the natural tooth, the portion of the exposed labial surface to be enamelled is marked with a sharp instrument. The crown is removed, and the part within the mark thinned with a corundum-wheel, and then perforated at regular spaces. The crown is readjusted, and the portion to be enamelled bent with the burnisher anywhere against the surface of the natural tooth. The crown is then removed, and moistened glass enamel, which should be moistened with distilled water, placed over the indented surface in quantities sufficient to cover it. The crown is then placed in a muffle, made of thin platina. The inside is coated with whiting heated, and the surplus whiting brushed out, so there will be no adhesion of the gold to the platina. With a flame directed towards the closed end of the muffle, it is heated to a point sufficient to fuse the enamel. The crown is removed when cool, more enamel applied, and the crown again baked. Next, it is ground with a corundum-wheel, and the surface levelled, and flaws or pits opened up. These pits are then filled with the enamel, and the crown again baked. After this the enamel is ground to the level of the crown. The enamel and gold are then finished with sand-paper disks. A thin mixture of the enamel is applied with an artist's brush, and the final baking given, which should be at a high heat, as high as the gold of the crown will permit. The crown should be formed of gold alloyed only with platina, and then the heat can be raised to as high a temperature as will melt pure gold, and you will not melt the crown. The open end of the muffle should be turned towards the operator, so he can see the exact amount of heat applied. I feel rather modest in presenting this to you, because I have not been able to give it a very great test, but from what I have seen of it, and from what the gentlemen who have examined it seem to think of the method, there may be considerable in it. I had Dr. Ames fracture the front of one of these models, to show how strong it is, and he says it is about as strong as a piece of porcelain enamel could be. Here is another model which is perfect. It is a little discolored, because there has been a great deal of solder placed in the crown. This one that was fractured by me was enamelled in twenty minutes at the Maryland State Society. In the mouth, the strength of the adhesion of the gold is very good. This piece is clinched on the inside. In the final operation of the enamel, I place with an artist's brush a little on the inside of the crown, so that it keeps firm. It leaves an additional amount of the enamel at the occluding surface, which forms quite a strong base for the piece of enamel which forms the front.

*Dr. Thompson.*—Does it keep its shade perfectly?

*Dr. Evans.*—I apply the enamel in successive layers. Each time I bake down and thoroughly fuse, at a very great heat. In the final one I give it a contour, grind it just as I want it, and then with an artist's brush I enamel over the surface, giving it a very fine coating, and then I give it the final baking at a high heat. The material I have used is that of Timme & Co. They have a variety of shades, and some directions in regard to producing different other shades, so you can get any color you like. This is less objectionable to many than gold, but I do not think it will be less objectionable than teeth which are filled with amalgam. People will tolerate a tooth in that condition where they would not a gold crown.

*Dr. Patterson.*—We have been looking for something of this character for some time. I would like to ask how the doctor controls the heat so as not to melt down the gold crown, and yet get it to the proper fusing-point. It seems to me to be rather difficult to withdraw the heat at the proper time without injuring the shell.

*Dr. Evans.*—That is very easily answered. I turn the open end of the muffle towards me, and direct the heat to the back of the muffle, so I can see my crown. It will be necessary for a person to make a crown or two to practise, and carry the heat to such a point that he will actually melt the crown, so he will know how high he can safely go. If you undertake to enamel one for a patient, the very first time you will probably melt it. You must practise first. I protect it from the carbon of the flame. The one at the clinic was done with an ordinary lamp with an old-fashioned blow-pipe. I did not wish to put the gentlemen to any trouble, so I carried my lamp with me and did it there. With an alcohol-lamp there is less danger of carbonizing your enamel than with an ordinary blow-pipe. You must blow a strong blast so as to effect a perfect combustion of the carbon of the flame, otherwise you would very quickly carbonize the enamel and spoil it.

*Dr. Peirce.*—What preparation of platina would you use?

*Dr. Evans.*—From three to four per cent. of platina in pure gold.

*Dr. Barrett.*—Wherein does this differ from the process presented by Dr. Timme?

*Dr. Evans.*—I did not see his process, but Dr. Timme has been unable to produce any such result, and they have been very anxious to find out how I did it. Dr. Timme simply takes a crown which is alloyed considerably with copper, and, from what I have seen, he places it merely on the surface of the gold. That will chip off; it is not safe for the patient to have in the mouth, and there is no result in an artistic way. This is to overcome the objection there is to his method. The crown in this case is perforated, and my piece of enamel is anchored through the crown and fastened on the inside. I have not seen it before, and I do not think it has been published in any journal. The man who has presented a method almost similar to this is Professor Littig, of the New York College of Dentistry. It will be found in the reports of the New York Odontological Society. He removed the labial surface of the gold, placed a fine layer of platina, and then put on his glass material and baked it; the merit of my process is in its simplicity. There is no soldering to be done. I thin the gold instead of cutting it out, and then perforate it. He put his material on, and it was not anchored through the platina, and it was liable to chip off. He did not fuse it in the way I have done, in successive layers. My process is to anchor it through, fasten it on the inside, put it on in thin layers, and polish it just as you want, and not have it bulge out like Dr. Timme's. His stood out beyond the other teeth, like some crowns which stand out. The result is anything but artistic.

*Dr. McKellops.*—I saw this brought out at Berlin by Dr. Cohnheim, of Liverpool. He was doing enamelling work of this kind. He was putting it on plates, and I would like to ask Dr. Evans if this enamelling is a secret, or does he give the manner of making it.

*Dr. Evans.*—It is not my enamel; it is Timme & Co.'s. There is nothing secret about it.

*Dr. McKellops.*—I was in Berlin at the time, and Dr. Cohnheim gave a demonstration there of different plates. He gave different colors, put a gum color on, as it were, and he had several specimens. He put it on almost anything. What the stuff was made of I was unable to ascertain. He said he would give it to me, and I told him I was in the habit of going among the profession in this country, and that I had some experience in using enamel. I could not get the secret. He said it accomplished the same result on gold or platinum, or, in fact, anything else.

*Dr. Evans.*—If this is a question of priority or honor, I think we must go back farther than that; we must go back to our friend, Dr. Herbst, who visited us a few years ago. He introduced a method from which I got my idea of the muffle. He took glass buttons and various things, and formed these enamels. They are only compositions of glass. He took the steel thimble he had and produced the same result that I produced to-day. I am introducing it to you to advance the subject of prosthetic dentistry. It is all mechanical, and you may bring your results as you wish, and place them as you wish, but to Dr. Herbst belongs the honor of this little muffle, and a good many other things.

*Dr. Guilford.*—The credit of this kind of work belongs to Dr. Herbst, of Bremen. When his brother George was in this country, some eight or nine years ago, he exhibited to me a method of grinding or pulverizing crimson glass, and fusing it upon the neck of a tooth to imitate the gum color. He pulverized the glass to a fine powder, and used it on a tooth that was unusually long, and he did not care to have the whole of the white tooth show. After it was crowned, he moistened it with water, sprinkled the glass on it, put it in the muffle, and fused it on. He did not make a very fine color, but it was better than white. The different processes used to-day have originated from that method. They were nothing more than glass beads. You can buy them and pulverize them. Glass is more fusible than porcelain. It is not as strong, but you probably could not use porcelain on a gold crown. The reason this can be melted is simply because the gold is alloyed with a certain percentage of platinum. Gold fuses at a degree of two thousand.

In regard to the platinum crucible, I got the idea from Dr. Herbst. Any one can make it, if he wishes. Take a piece of thin platinum or iridium, having it the proper length; bend it over the finger and place the edges together; then put on a little gold, and you have a muffle that is entirely perfect. Where Dr. Evans lines his platinum muffle with a layer of whiting, I have been in the habit of using a thin piece of asbestos-paper or felt. It prevents the contact with the gold, and gives you more general heat. One thing more I will mention in connection with the heating of muffles in that way. We all know that sometimes it is advisable to do a little soldering to a plain tooth, without taking the time to harden it. It can be done very readily, as, for instance, where we have a plate tooth and want to convert it into a tooth suitable for rubber work. It can be done by grinding the pin flat and laying the gold across it. Put it on the asbestos-paper, and slip it into the muffle, and it will heat at once. There is no danger of cracking the tooth, and, after you have heated it, just tip your muffle and let the tooth slide right out. I have done it repeatedly, and never cracked a tooth. It is a very ready way of uniting the pins of a tooth under different circumstances.

In regard to glass filling, which would come up here incidentally, I feel that many persons are interested in the matter. They promised a great deal in the beginning, but I do not think they have held out. A glass filling at best does not look very well. It looks better in the front of a tooth than a gold filling, but the difficulty of producing a proper shade, and of setting it in the tooth so the margins shall be good, is extremely difficult, if not impossible. It is better than nothing; but when we come to make a glass filling, building up the corner of a tooth, etc., we get on dangerous ground. I had a sad experience, and I think it is well to warn men in regard to them. They lack the quality of strength. In the centre or the face of a tooth no pressure is brought to bear, but on the corner of a tooth you will find they break. If they are placed, as Dr. Evans does his, in a muffle and melted down successively, you will get better results; but from the careless way in which it is sometimes done, you get only a weak mass, and if you place it where there is great strain, ten to one your operation will fail.

*Dr. McKellops.*—As far as enamelling is concerned, the originators of that method were watch-makers. That is where we get it.

Subject and Section passed.

### Section II.

Dr. Ottofy made a report, an abstract of which follows:

"It has been the custom of the Section annually to report the number of dental colleges in active operation in the United States. At the close of the last session the number was thirty-three, there having been no increase over the previous year. There have been established since last year the Dental Department of Tennessee

Medical College, at Knoxville, Tenn.; the Chicago Tooth-Saving Dental College, at Chicago, Ill.; the Dental Department of the Homœopathic College, at Cleveland, Ohio; the Dental Department of the Western Reserve University, at Cleveland, Ohio; and the Dental Department of the University at Buffalo, making an increase of five over the previous year. The total number now in operation is thirty-eight. The number of graduates in 1891 was twelve hundred and forty-one; in 1892 there were fourteen hundred and eighty-three. The average in the last seven years is nine hundred and four graduates per annum. The superfluity of many of these colleges was well demonstrated last year, when attention was called to the fact that it required the strength of fifteen colleges, more than one-third of the number, to graduate less than one hundred students; eight more turned out one hundred and fifty, while twelve colleges out of thirty-three graduated more than three-fourths of the entire number. We have here a tabulated statement of all the dental colleges. Two of the new colleges have held no commencement. It required this year the force of fourteen full-fledged colleges, with their equipments, Faculties, etc., supposed to be perfect, to graduate ninety-one students, an average of six and one-half each. In fact, eleven colleges together graduated fifty-one students. Who can estimate the terrible loss to the profession, were eleven colleges to be suddenly taken off the list? Eight hundred and fifty-one were passed from ten institutions, or a little more than one-quarter of the entire number of colleges graduating almost two-thirds of the list. The three years' college course does not seem to have had the ill effect so strenuously predicted. On the contrary, the number of students entering the colleges, though smaller, is superior. They fully appreciate the additional advantages gained. The number of years which a student should devote to dentistry will soon be placed at four years of nine months each. Any young man with a proper preliminary education will find that time sufficient to become proficient. There are now about one hundred and thirty local societies in the United States, with an aggregate membership of nearly five thousand.

"The Executive Committee has issued circulars to all the societies with a view of increasing delegate membership to this society. Two years ago twenty-two, and last year twenty-four, societies were represented in this Association. There are not as many this year.

"One of the important advances made in dental education during the year is the establishment of reading courses, somewhat after the Chicago idea, on the part of the Post-Graduate Dental Association. It comprises several reading courses of from two to five years. No two are alike, and any one can profit by reading in several classes at the same time. The courses cover a wide range of reading, touching on the border-line of allied science. A certificate or degree will represent exactly what it says. None of these courses are to take the place of the college course, but are merely to supplement it, and to imbue the dentist with the scientific, literary, and artistic aspects of dentistry.

"Several additions have been made to the literature during the year. The periodical literature is far superior to that of a few years ago, and it is equal to that of the medical profession. There is to-day but very little copying done, most of the contributions being original. In the report of the Committee on Dental Science before the State Society, the following recommendation was made: 'That a good quarterly journal was needed, filled with nothing but original matter, edited by a competent dentist, and published by a house selling dental goods.' In addition to this, some one should start a weekly dental journal of sixteen to twenty-four pages, in a central location, so that dentists could be kept *au courant* with dental news. We think that it would pay. The weekly could be sold for three dollars per year, and if ten thousand subscribed, there would be a great awakening of interest.

"The following have been added to the literature:

"'Harris's Dictionary of Dental Science,' which is very valuable.

"A new edition of Sewell's 'Dental Surgery.'

"'Charts of Typical Forms of Constitutional Irregularities of the Teeth,' by Dr. Talbot, has been published; also a second edition of 'Descriptive Anatomy of the Human Teeth,' by Dr. Black.

"'Catching's Compendium' has also been published, and is very valuable when it is desired to find several articles on the same subject.

"The most recent contribution is the work on 'Dental Jurisprudence,' by Dr. Rehfuss.

"This concludes our report."

*Dr. Hunt*, of Iowa.—There is still another school that has issued a degree which is not mentioned in that list; it is in Alabama.

*Dr. Patterson.*—There is also one in Cincinnati.

*Dr. Allport.*—I do not wish to say anything against any reputable college, but it seems to me that what is termed "tooth-saving college" ought not to be dignified with a mention in this section. It should not go into our report, and I move that that part of the report be stricken out.

Motion seconded.

*Dr. Peirce.*—I hope it will not be stricken out. It is simply a record.

*Dr. Truman.*—I desire to endorse Dr. Allport's idea. I think it is wrong to have a name of that kind go out into the world in our reports. Many judge character from the stand-point of names, and this title "tooth-saving college" is certainly sufficient to condemn us.

*Dr. Horton*, of Cleveland.—This Society does not claim to endorse the reputation of any college or any institution. The chairman of the Section has given us the facts. I do not see that we render ourselves ridiculous by simply accepting the facts, without any endorsement. The fact is that such a college exists.

*Dr. Ottofy.*—I admit that I am ashamed to have been obliged to report such a college, but, as has been stated, it is a fact. It grants degrees, and there are sections of this country where the degrees are recognized. Many of the schools which are good to-day were, eight or ten years ago, just as bad as that one; consequently, this is a fact. The college is in existence.

### ORGANIZATION OF SECTIONS.

*Dr. Harlan.*—The secretary is required at this time to read off the list of the Sections, and take the names of those members present who desire to join them, and I will now proceed to do so.

Adjourned.

### *Second Day.—Evening Session.*

The meeting was called to order by the President, Dr. Walker. Dr. Shepard read the report of the Committee on the President's Address, an abstract of which is as follows:

"The committee to whom was referred the address beg to submit the following as their report:

"With reference to the suggestion relating to the World's Fair in 1893: Recognizing the importance of the Congress, and its joint parentage in the American and Southern Associations, as set forth in the address, we recommend that an appropriation of five hundred dollars be made from the funds of the society to the treasurer of the World's Columbian Dental Congress. In this connection we are pleased to state that at the recent meeting of the Southern Dental Association an appropriation was similarly made for the same purpose.

"DENTAL EDUCATION, LEGISLATION, AND THE DENTAL DEGREE.

"Your committee fully coincide with the sentiments of appreciation of and confidence in the work which is being done for the improvement of dental education by the Faculties of a good proportion of our colleges. The unification of State laws is very desirable. Efforts have long been made to secure the same in matters at the foundation of society, as marriage and divorce, and at the foundation of business, as in insolvency, with results that we need not dwell upon. We can hope and expect, through means within our own control, especially by the strengthening of the societies in each State, that each board will be of such knowledge and efficiency that a certificate of examination will be respected by the boards of other States, and either take the place of or modify the examinations. In most States the scope and thoroughness of an examination is entirely within the control of its board, the only obligation being that the board shall be satisfied that the candidate possesses the requisite knowledge and skill to meet the State's demand of safety to its citizens.

"In reference to the suggestion, 'That all States should be divided into districts, each having a board of examiners, and in the State board each district shall be represented by one examiner, and all boards so constituted shall be elected by the dentists of the State, and never appointed by the governor of the State,' we would answer: The question of the appointment to examining boards is surrounded with great difficulties. There is no justification for a board at all, except under the police powers of the State, and as safeguards for the health of the citizens. The members of boards are officers of the State, sworn to support it and faithfully perform their duties. Their power is derived from the State, and after appointment must be from the State, either directly or indirectly. There is no perfect government either in its plan or execution; every good citizen should aim to find out the best, and aid in the evolution of the perfect. The objection to an appointment by the governor is that he is not likely to know the most competent men, or will be swayed by politicians and political motives. Suppose in any State the State society embraces in its membership every reputable dentist, or even eighty per cent. of them. Would it not be feasible to get the consensus of the views of the members of the society as to the proper appointee, and by any suitable method recommend to the executive a candidate? Would not an intelligent governor welcome such a system and be glad to be thus relieved of the responsibility, and would not a political governor realize the political power back of such petition, representing most of the dentists and their influence with their patients who are voters, and consider such an appeal to him as paramount to, or vastly greater politically than, any similar petition coming from any source whatever? In this way the objection to lodging the appointing power in the governor is greatly lessened, and, besides, presents a less complex system of securing a competent board than the one suggested.

"We respectfully submit that it is an ambition which all should share; that matters should be so managed in each State that the State society shall practically embrace the reputable profession in such State. This we regard as the principal factor in securing the best laws and their proper enforcement, the most competent board, and the much-desired interstate courtesy and recognition of State licenses.

"COMMITTEE ON STATE AND LOCAL ORGANIZATIONS.

"With reference to the formation of this committee as suggested by the president, your committee, realizing the benefit which the work of such a standing committee would be, not only to this Association, but to the whole dental profession, and coinciding with the spirit of the plan, offer the following resolution:

"*Resolved*, That a standing committee, to be styled the Committee on State and Local Organizations, and composed of three members, shall be appointed originally for terms of one, two, and three years respectively, and that all vacancies due to expiration of term shall thereafter be filled for a term of three years. All vacancies occurring from any other cause shall be filled for the unexpired term. The basis of the plan and scope of work of said committee and its duties with relation thereto shall be the suggestions as set forth in the address of the president of the American Dental Association for the year 1892, also the articles from which he quotes, and in harmony with the circular letters issued by the chairman of the Executive Committee.

"In regard to the meeting of the American Dental Association for 1893, we recommend the adoption of the following resolution:

"*Resolved*, That the annual meeting of this Association for the year 1893 be convened at the time and for the purpose suggested in the president's address, and that the consideration of the report of the special committee embodying certain proposed amendments to the constitution and by-laws be laid over for consideration at

4

that meeting. We recommend, also, that the matter of the death of Dr. John Allen, of New York, be referred to the Standing Committee on Necrology for proper action.

"Respectfully submitted,

"E. C. Kirk,

"L. D. Shepard,

"J. N. Crouse,

"*Committee.*"

Report received.

Dr. Truman requested the privilege of the floor for a few moments on a subject not strictly in connection with the business before the Association. This being granted, he remarked:

"I have been impressed for a long time with the changing character of human life. The constant mutations are always a subject for thought, but as individuals grow in years, and the time comes for the closing of life's activities, we necessarily feel these more deeply if they be complicated with financial reverses. During the past few months one of the most distinguished members of our profession in the United States in past years, one who stood probably higher than any man in this room stands to-day, one who was looked up to by all, is now an inmate of a Masonic home in Philadelphia, and to-day some of us find it necessary to aid a fellow-member of this Association, one whom we have all honored, and whom I knew very well when I was but a stripling in the profession of dentistry. He has done more, perhaps, for its advancement than any man in the past, and probably more than any one now in this audience. I allude to Dr. W. H. Dwinelle, of New York City. The 'parting of the ways' has certainly come to that life; he stands to-day with a clientele of exceptional character, and yet with present poverty staring him in the face. To-day Dr. Dwinelle has not one dollar to call his own. It does seem to me that when such a man, who has done so much for us, reaches this period, no matter what may have occasioned it, no matter that he may possibly have been improvident, we should do something in return. Dr. Dwinelle is at present living at his former home in Casanovia, under the care of a sister, and will remain there possibly until the final close of life comes to him, as it comes to all of us, sooner or later.

"In view of this fact a few members of this Association have come together and appointed a committee to take charge of any fund that may be realized. A few in New York City raised a preliminary sum for his benefit. It is now proposed that two funds be provided,—one to which any one may subscribe, to give a certain minimum sum, say five dollars yearly, or more if they see fit; and another in which they may, if they prefer, give any amount they please, and so finish up the matter, but preferably that they would give a certain sum every year. This will be placed in the hands of a committee which consists at present of Dr. Perry, of New York, Dr. Allport, of Chicago, and myself in Philadelphia, who will see that this is properly given to the recipient. All I want to do this evening is simply to call the facts to mind. Dr. Dwinelle is perfectly willing, after explanation, that this be done, and he would regard it as a testimonial to his honor, and to the work that he has accomplished. Therefore it is no degradation to him, and I feel it a pleasure as well as a duty to stand here in your presence and talk of this man whom I have respected all my life."

*Dr. Allport.*—I am very sorry that I have to give my services in this direction, but at the same time I shall be very happy to do anything I can for our old friend, Dr. Dwinelle. In regard to this matter, I was told what they had been doing in New York. I said, "I cannot consent to allow New York to do all there is to be done." He belongs to us all; he was not only a father to the younger, but also to the older practitioners in this country, and as our good friend Dr. Truman says, probably no one has done more than Dr. Dwinelle in his earlier days for the advancement of dentistry. I remember, when I commenced, that my preceptor used to take the old *American Journal of Dental Science*, and I never opened that journal without looking for the name of Dr. Dwinelle, of Casanovia. I felt that if his name appeared the article would be interesting. I was speaking to Dr. Morgan, and he mentioned a certain article; I remember the exact place I stood when reading it, in the little town where I then resided. I have never gotten over the impression it made upon me. Perhaps Dr. Dwinelle and Dr. Dunning, of New York, impressed me more strongly with a desire to succeed and make myself useful than any other two men who have ever lived. I remember distinctly an operation of Dr. Dunning, that has been before me for fifty years. I carry it in my memory as vividly as when I looked upon it, and I try to do as well as Dr. Dunning did. We are greatly indebted to Dr. Dwinelle, and it would be a shame to every man who has any means who is not willing to contribute his mite to help our good old friend in his trouble.

I was never impressed more with the importance of dentists taking care of their money than from what I heard to-day. Look at Dr. Atkinson, at Dr. Maynard, at Dr. White, look at Dr. Dwinelle,—all men of eminence, men of great ability,—and see the condition they arrived at. Let me say one thing to you now,—take care of your money. Don't be mean or niggardly, but don't spend it without you get value for it. Don't squander it!

*Dr. Morgan.*—I had not intended to say anything to-night on this subject, but some of my friends insist upon my doing so. My early impressions of the character of Dr. Dwinelle are very vivid. When I first learned that there was such a journal as the old *American Journal*, as we call it now, I lived away in the backwoods, and had a preceptor who did not know there was a journal like that in the world until I stumbled upon the fact and wrote for the papers, and they sent them to me. I found the articles of Chapin A. Harris, and also articles signed "The Casanovia Editor," and without knowing who he was, and in my ignorance of the profession and of the qualifications often necessary for its successful pursuit, I began to read these articles, and they made a profound impression upon my mind. I learned to admire them for the clearness with which the author stated his propositions; for years afterwards I always hunted up his articles first. In speaking to Dr. Allport, I recalled to his mind an article that appeared in 1847, upon a subject of the treatment of teeth where there was deep decay, and I found a notice among my papers quoting what he said, what he did, the date on which he said it, and the fact that I proposed to follow his ideas. It was in April, 1847. I allude to that to show how his writings impressed me at the time. One of the best articles that journal ever had was the one I allude to. From then until the present time I have only lived to admire him and learn from him. His contributions have always been acceptable, and occasionally a patient of his fell into my hands, and I can say that the most beautiful operation I ever saw made by him with sponge gold was performed for Miss Nelson, a niece of the famous Nelson of the British navy, whose history you know.

Misfortune has overtaken our friend, as it is possible it may overtake any one of us. While he has made large amounts of money in fees in his life, in his old age he is poor, and I cannot see how any man, who has any humanity in him, does not have his heart go out in kindly feeling towards him. The highest attribute of humanity is the love of your fellow-men. It is perfectly natural for us to love those who are near to us, and it has been said that I love my family first and the dentists next, every one of them, without exception. I feel exceedingly kindly to this good man, who did so much just at the time that dentistry was merging into a profession, for that was the first literature we had in this country that was worth anything. Through that, and out of that, has grown all that we have in the line of periodical literature, and none has been more useful to the profession. *The American Journal of Dental Science* had its origin when he was a member of the old American Society of Dental Surgeons, and afterwards passed into private hands. It ought to be our privilege to do something to mitigate the evils of poverty in this his old age, and thus allow him to gently pass towards the dark river. That period will come to us all some day, and in talking about it to-day, I looked around and remarked that this institution of which we are members is growing in years. Taft, Allport, and Morgan are all that are left who were here at the organization; McKellops was here when it was put on its feet, but he was not present at that meeting. Only one other man is now living whom I can call to mind, who was there, and that is Flagg. A few of us are not very useful any more. We may enjoy life as well as we ever did,—I hope every man in this audience does that,—but we have not much longer to be in this world, and if we have any of its goods, it is but rendering obedience to the principle of the Master, to the promptings of sanctified human nature, to reach out a helping hand to those who are in need, especially those of our own household, and I consider the dental profession part of my own household. Let us help our old friend in his last days, and relieve his mind of the anxiety that naturally preys upon it. You cannot spend the same amount of money in any other way that will bring you a larger reward. A wise man uses this world so as not to abuse it. No professional brother can use the same amount of money to better effect than to give it to Dr. Dwinelle.

The subject was then passed.

Dr. Smith then reported on behalf of the Committee on Credentials, giving the names of the different institutions from which delegates had been sent, in all numbering sixty-three.

The Auditing Committee requested further time for their report.

The secretary read a communication from Dr. Frank M. Odell, in which he states that he is unable to attend, and offers his resignation as a member.

Dr. Taft moved that the dues of Dr. Odell be returned to him. He is not present with us to receive any enjoyment from this meeting, and I think it but just that this should be done.

Adopted.

### SECTION III.

### OPERATIVE DENTISTRY.

Dr. McCandless, secretary of the Section, reported as follows:

"During the year closing July, 1892, there have appeared in the different journals about sixty articles, among them the following:

"'Porcelain and Glass Inlays;' 'Extirpation of the Pulp,' 3; 'Treatment of the Pulp,' 4; 'Operative Dentistry,' 4; 'Capping Exposed Pulps;' 'Treatment of Pulpless Teeth,' 5; 'Relative Merits of Filling-Materials,' 4; 'Preparation of Cavities'; 'Art *versus* Nature, in the Form of Contour;' 'A Genesis of the Contour Filling;' 'Care of Children's Teeth,' 5; 'Vitreous Fillings;' 'Soft Gold-Foil as a Filling-Material;' 'Gutta-Percha;' 'Failure of Dental Operations;' 'Relative Advantages of Cohesive and Non-cohesive Gold;' 'The Cervical Border,' 2; 'Relative Conductivity of Heat by Filling Materials,' 2; 'Cocaine as an Agent in removing Pulps;' 'Care of Teeth during Pregnancy;' 'Value of Crystal Gold in Dentistry;' 'Inoculation from Unclean Instruments,' 2; 'Combination Fillings,' 6.

"I have in my possession the memoranda of all these papers, and if any one desires to see the same, I shall be pleased to give all information.

"One article only has been written on capping exposed pulps, while many of our classes at college took as a text for their theses 'The Treatment of Exposed Pulps.' At most conventions the essayists who dilated on this subject have invariably been young men in the profession and in the practice of dentistry, so that my observations differ materially from those of the gentleman who made a report of this Section a year ago. The older members have less and less to say each year on the subject.

"Dr. Conrad promised an article on the subject of filling teeth, but he is not present. Dr. McKellops can say something in this connection, as he has been heard to remark that a man could fill a tooth with gold better if he had more than one instrument to fill it with. Drs. Harlan and Crawford can also say something about this.

"This society was unfortunate last year in not having time to hear a paper on 'Art *versus* Nature,' and one by Dr. Crawford on 'Children's Teeth.' They appeared in the dental journals, however, and probably had a much wider circulation in that way.

"No new work has been published on operative dentistry this year. The chairman of the Executive Committee, Dr. Crouse, has made a strong effort to bring the State and local societies into closer affiliation, and to that end he distributed circulars, requesting gentlemen to send him reports of the proceedings of their meetings, if there was anything of importance to the profession. To this he received twenty-five replies. In some instances, a fairly good synopsis of the proceedings was given, but in most of the cases the chairman failed to grasp the idea, and sent simply a list of papers read, with the names of the authors and the list of the officers elected. In reading over the various replies, I find little or nothing of interest. There is reason to hope that more thought will be given to this idea by the societies in the ensuing year.

"Dr. Ames has evolved the oxyphosphates of cement during the past year. One of the best cervical appliances is Dr. Gibbs's. He has given it additional strength by removing the front piece, and a firm spring holds the clamp in place until the thing is set. They can be had at any angle, to suit any case.

"A sand-paper disk-holder that cannot be improved upon has not been discovered. There are improvements in Johnston's cervix-clamp, Dr. Howe's heater for softening gutta-percha, and modifications of the Kells electric holder and improved root-pliers. Among the new devices are Bosworth's universal mallet, and the silver and platinum alloy screw-holders.

"Two papers have been prepared for this Section,—one on 'Transplantation,' by Dr. Morrison, of St. Louis, and one on 'Matrices,' by Dr. Cravens, of Indianapolis, Ind."

Report accepted.

Dr. Morrison, of St. Louis, Mo., then read a paper on "Transplantation," an abstract of which follows:

Of the cases reported in 1875 or 1876, very few are now to be seen. One of these cases, Mrs. B., was constitutionally the most unfavorable subject that could have been selected. While a majority of the others were favorable subjects, yet their teeth dropped out after a few years with their roots resorbed.

In many cases the sockets were not favorable to plant others, or the patients would not submit to the operation again. The manner in which these operations are regarded makes it exceedingly unpleasant for an individual who performs the operation. For example, a Mrs. H. B. had a left superior bicuspid implanted in November, 1887. Being the daughter of a physician, the matter was talked over from all stand-points, and she consulted the profession, both medical and dental, of this country and Europe, without getting one good word in favor of such operations. On April 21, 1892, it was with difficulty that she was persuaded to have another implanted in its place, as one had already been in there. For two or three weeks she had all kinds of unfavorable symptoms. Erysipelas was threatened, and it was thought that she surely had that disease; then blood-poisoning, fever, loss of appetite, loss of sleep, etc., ensued. I was not alarmed at any time, but my patient and her husband and family were. I had seen similar symptoms with no bad result. Her physician was in my favor, and told me not to pay any attention to her complaints. He knew her disposition. If that patient had died, or become seriously ill from any other cause, I would have been held accountable to the law for performing an operation not endorsed by the profession.

Another case was that of Mr. E. C. K.; left superior second bicuspid. It was implanted in a socket. The face was much swollen at the time of the operation. Considerable pus oozed from the socket. The swelling continued for eight or ten days, and he complained of peculiar sensations under the eye and towards the ear. He made several visits to have it extracted. The patients seemed to be entirely unhappy with the teeth in the mouth for a day or two, and came from day to day to have them extracted, but I would not take them out.

Another case: A superior lateral incisor had been planted by a neighboring dentist, but he used a very dry specimen and drilled a large hole to fill the pulp-canal with gutta-percha, and the swelling of the gutta-percha by moisture, and the drilling, had caused about an eighth of an inch of the crown on the labial half to come away. The remaining stump and root were healthy. I adjusted a gold crown, which was worn but a short time when the rest of it broke down. I removed the remainder of the root and planted another dry tooth in its place, and the gentleman went away to an adjoining city. I supposed it was not successful, but he returned and I found that that tooth was doing very nicely, and in a perfectly good condition.

Mrs. McK. had two teeth implanted, the first bicuspid on each side, on May 31, 1888. The root became resorbed, and I had to implant another in April, 1892.

Mr. E. W., aged about thirty, had never erupted the left superior canine. For several years he had been waiting for it; finally it appeared, but only enough to be cut down, and extracted from a horizontal position in the mouth just inside of the bicuspids. The tooth was embedded in a horizontal position. It was a large, well-formed tooth, except for a crack near the end. A deciduous canine was now extracted from its normal position in the jaw, the socket prepared, and the permanent canine that I took from the roof of the mouth was implanted in the position where the deciduous tooth was removed. It has grown perfectly attached to the socket.

Mrs. F. E. A. had a right superior first molar, three distinct roots, implanted November 2, 1891. Healed well and never gave a moment's uneasiness.

Miss C. had a lower incisor treated for months for catarrh. It was finally pronounced incurable, and was extracted, making the first space in the lower arch. As the patient was an artist, it annoyed her very much, and after three weeks she appealed to me for relief. I implanted another in its place. It was fairly firm.

To attend ordinary dental meetings and hear papers and discussions, one would think every operator would have no difficulty in preserving teeth by placing a small amount of gold in the tooth, and thus secure it for all time. From the mouths of the higher order of patients teeth are extracted, spaces are left vacant, or, worse still, ghastly porcelain counterfeits are placed in, which work mischief to the adjoining teeth. The slaughter-houses are not alone responsible for the toothless specimens too often seen. It has been my practice for years to transplant teeth to all sockets having good alveolar processes, where I remove a single root or two. With me the process is indispensable, and since Dr. Younger has given us the dry specimen, it is much more convenient, although the fresher the specimen, the better. Like a broken bone, security of position and absolute rest are essential. I use a copper wire ligature from the crowns of the adjoining teeth, under or over, as the case may be, and over the edge of the implanted tooth, also securing the ligature in position, and the interdental space, with phosphate cement, which is worn for six or eight weeks. This ligature is a very excellent thing. I rarely use silk or hemp. I formerly used platinum, or else rubber plates to be removed by the patient, but a neater process consists in making it of one piece of wire encircling the tooth, joining it on each side, and then twisted at the most convenient point, making a figure eight. Then join the free edges of the copper wire and the interdental space with phosphate cement, allowing the retention to be made perfectly secure while it is in that plastic condition.

Dr. Hunt then read the paper of Dr. Junius E. Cravens, who was unable to be present, entitled "Matrices."

It is a test of an operator's judgment sometimes to determine whether he shall use a matrix, or merely a support for his filling-material; there is a wide difference.

Webster's Dictionary says that a matrix is "a mould; the cavity in which anything is formed, and which gives it shape." If this definition is correct, not everything which dentists build gold against should be dignified as a matrix. A neighboring tooth often serves the purpose of a support, but who would regard a supporting tooth as a matrix?

A matrix for filling should offer certain advantages, such as thinness for economy of space, close conformity to cavity margins, however irregular, sufficient malleability to enable the operator to bend and dilate at any part and to any extent desirable; it should be cheap enough to be thrown away after once used.

Some proximal spaces are impossible, of course, but extensive preliminary wedging should never be necessary to adjustment of matrix or support; otherwise the device is too thick and badly designed, and the operator's judgment is at fault.

Some operators, perhaps many, use closely adapted matrices for gold filling in compound proximal cavities in bicuspids and molars, and it is not my purpose to criticise their methods, because of lack of sufficient opportunity for observing results; but criticisms have recently appeared in some of the dental journals, one writer going so far as to declare that perfect gold filling cannot be done in matrices. I think he is mistaken, for every cavity is a matrix so far as it affects the form of its contents; but from the few cases from the hands of others that it has been my privilege to observe, and a fair number of my own, I am satisfied that failures may easily be made, and that an operator's confidence in matrices sometimes is abused, because failures at obscure margins may not be discoverable until after the completion of the work.

The usual fault with matrix filling consists in imperfect adaptation of filling-material at cervical and lateral margins; for some operators to meet this objection may be easy, but there are those of us for whom it is not. Gold cannot always be depended upon for perfect adaptation at margins, unless in excess, and close matrices do not admit of excess.

By a support, as distinct from a matrix, is meant something that can be placed between two teeth, one or both of which may require filling, and serve to support the filling-material until secure, but without effect upon the contour. A supporter should also economize space between the teeth to the same degree as a matrix; nothing more than the slightest primary wedging is really necessary for adjustment of a supporting device of proper thinness. A supporter should be of metal, very thin, springy, non-malleable, with no attempt at conformation to cavity margins; on the contrary, the margins should stand free, particularly in filling with gold, in order that an excess of that material may occur there for subsequent dressing down. Gold cannot always be depended upon for correct adaptation at margins unless in excess, and close matrices do not admit of excess there.

Perhaps there are operators who can secure exact marginal fulness with gold, no subsequent dressing or finishing being required, but most of us find it necessary always to dress away some at the margins for security.

PLASTICS.

In filling with amalgam or cement in compound cavities in bicuspids and molars, matrices are advantageous; in fact, in any cases where contouring with plastics is intended; and reversing the rule for gold, the matrices for plastics ought to be closely adapted at all involved margins, and should mould the contour to any desired degree because the plastics are accommodating, and with careful manipulation a perfect cast of a matrix and approved marginal adaptation are attainable by all.

Matrices offering the advantages named in this paper may be constructed of German silver rolled to thirty-eight United States standard-wire gauge, a strip of which should be cut and fitted around the cervix of the tooth, and made to conform to the curves of the gum in the same manner as in fitting bands for gold crowns, etc., exercising care to avoid occlusion with teeth of the opposite jaw; a band should close with a lap-joint next to the cheek, and should be fastened with soft solder. In position, such a matrix may be dilated or bulged at any part, the metal yielding readily to moderately firm pressure, so as to conform to an approximating tooth or give fulness at any point.

Amalgam thus supported may with safety be permitted to stand several hours to acquire hardness, and often to accommodate the dentist's time demands. No wedging, no ribbon-saw, necessary, because the band has guaranteed enough space for finishing purposes, admitting the thinner strips and disks employed for that purpose.

In filling with gold in the same class of cavities, plain shields of the same thin German-silver will take and hold all the space required, and at the same time, if a little more space should be desirable for finishing purposes, it may easily be gained during the operation of filling by driving the gold firmly against the supporting shield, the gold actually wedging the teeth apart slightly.

I have made beautiful and durable contours with os-artificial by use of the band matrix, a fact which is merely mentioned here to show the scope of utility of the band device. The German-silver band is easily constructed, easily adapted to margins, and made to mould any vertical contour, and is cheap enough to throw away after being once used.

To remove one of these band matrices the lap-joint should be opened, although it often may be done without.

(To be continued.)

